# Supplementary material for: Spike-based dynamic computing with asynchronous sensing-computing neuromorphic chip
Source: Nat Commun. 2024 May 25;15:4464. doi: 10.1038/s41467-024-47811-6 (PMC11127998; doi:10.1038/s41467-024-47811-6)
Supplement: Supplementary file 1 — Supplementary Information [file 41467_2024_47811_MOESM1_ESM.pdf]

Supplementary Information for Spike-based Dynamic Computing with Asynchronous Sensing-Computing Neuromorphic Chip

Man Yao[1,†], Ole Richter[2,†], Guangshe Zhao[3,†], Ning Qiao[4,2,†], Yannan Xing[4], Dingheng Wang[5], Tianxiang Hu[1], Wei Fang[6,7], Tugba Demirci[2], Michele De Marchi[2], Lei Deng[8], Tianyi Yan[9], Carsten Nielsen[2,10], Sadique Sheik[2], Chenxi Wu[2,10], Yonghong Tian[6,7], Bo Xu[1], and Guoqi Li[1,11*]

[1]Institute of Automation, Chinese Academy of Sciences, Beijing, China
[2]SynSense AG Corporation, Zurich, Swizerland
[3]School of Automation Science and Engineering, Xi'an Jiaotong University, Xi'an, Shaanxi, China
[4]SynSense Corporation, Chengdu, Sichuan, China
[5]Northwest Institute of Mechanical & Electrical Engineering, Xianyang, Shaanxi, China.
[6]School of Computer Science, Peking University, Beijing, China
[7]Peng Cheng Laboratory, Shenzhen, Guangdong, China
[8]Center for Brain-Inspired Computing, Department of Precision Instrument, Tsinghua University, Beijing, China
[9]School of Life Science, Beijing Institute of Technology, Beijing, China
[10]Institute of Neuroinformatics, University of Zurich and ETH Zurich, Zurich, Switzerland
[11]Key Laboratory of Brain Cognition and Brain-inspired Intelligence Technology, Beijing, China
[†]These authors contributed equally to this work.
[*]guoqi.li@ia.ac.cn (corresponding author)

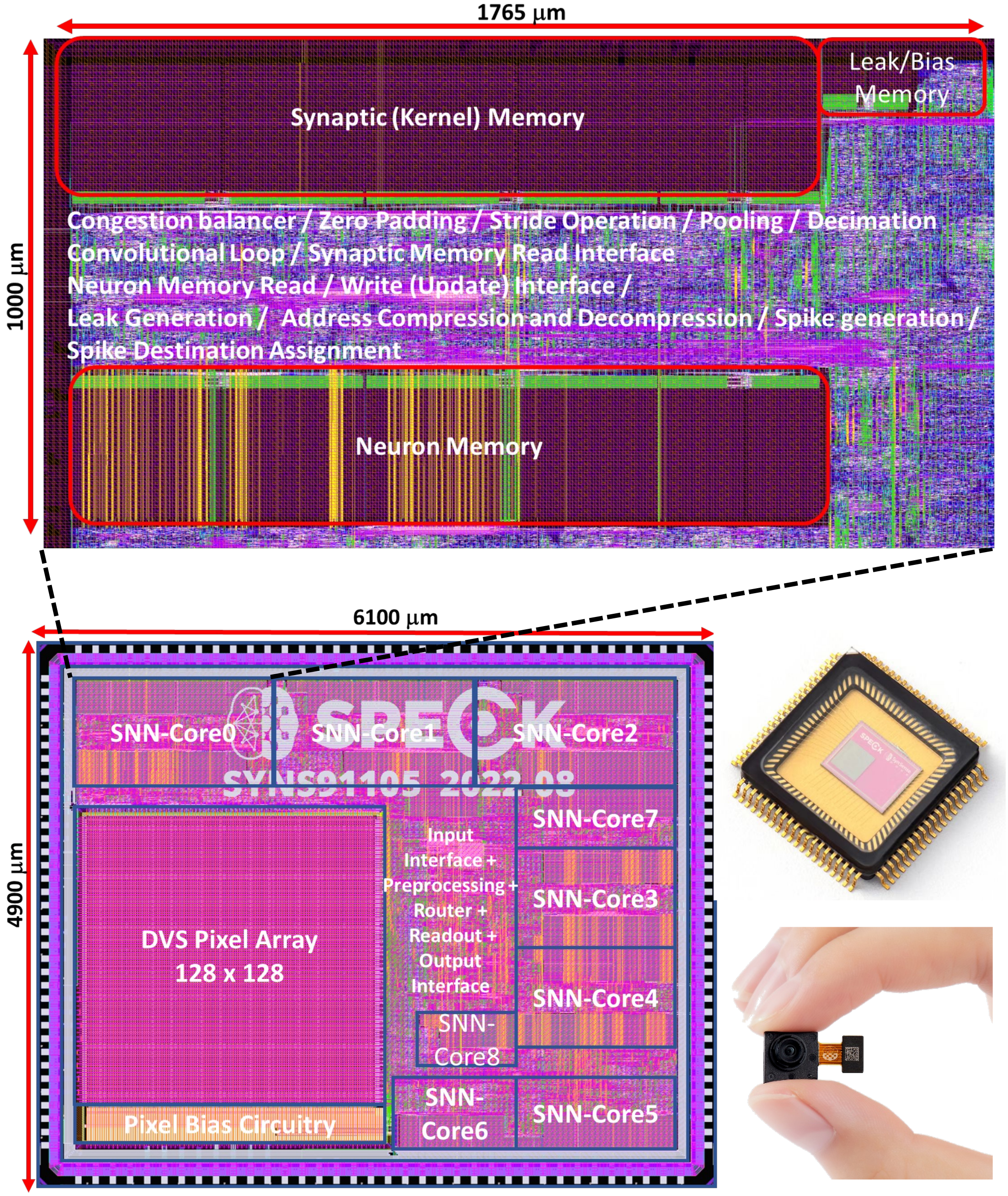

Figure S1. Fabrication of Speck. Top: Single SNN core layout with dimision of 1765μm × 1000μm, contains 3 memory block for kernel, neuron and bias, and multiple functional circuit designs. Bottom Left: Speck chip layout with die size of 6100μm × 4900μm, comprises with DVS pixel array, multiple interfaces and 9 SNN cores. Bottom right: (top) Speck chip packaging with CQFP80; (bottom): Speck Chip On Board (COB) packaging as optical module.

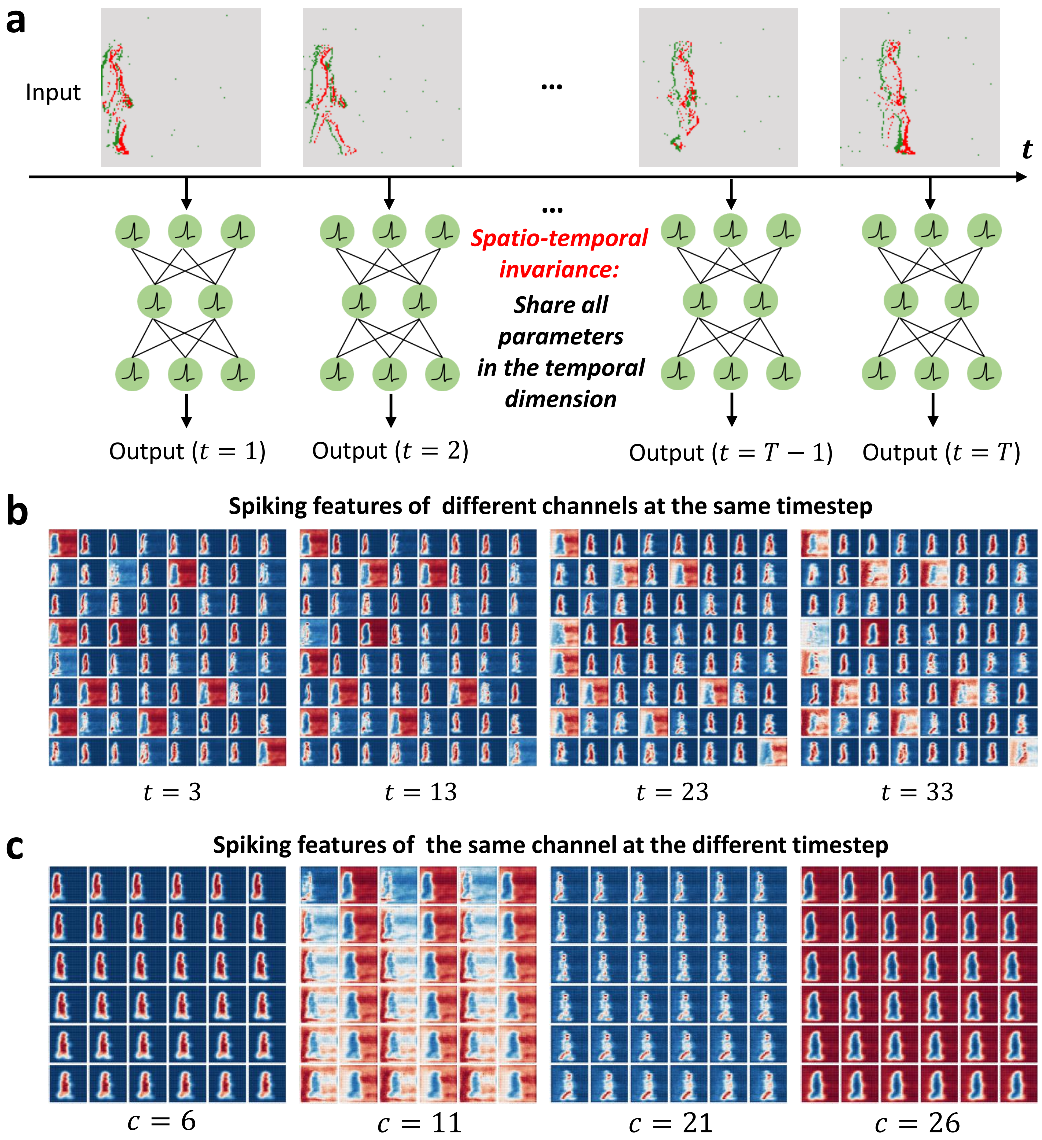

Figure S2. **Spatio-temporal invariance of SNNs.** **a**, SNNs exploit the same set of parameters for every location at each timestep. This fundamental assumption of SNN can be referred to by spatio-temporal invariance [1,2,3]. **b**, Visualization of spiking response features (averaging the output spiking tensor $\boldsymbol{S}^{t,n}$ over all samples, four dimensions: [T=36, C=64, H=32, W=32]) in vanilla SNN, where $t = 3, 13, 23, 33$ and $n = 1$ (i.e., first layer). We depict all 64 channels at each timestep. Each pixel in a channel represents a neuron's spiking firing rate over the entire dataset. For a single channel, the redder the pixel, the higher the spiking firing rate; the bluer the pixel, the closer the spiking firing rate is to 0. We can see that the parameter sharing in the temporal dimension leads to the closeness of the extracted spiking patterns at different timesteps. For example, the positions of the noise features (red channel) are basically the same at different timesteps. The main difference is that as the timestep increases, the human gait gradually moves to the right. In this way, it's easy to understand why the spiking firing rate of SNN at different timesteps is very close. **c**, Spiking features of the same channel at different timesteps. We can observe the impact of parameter sharing on feature extraction. For example, the extracted feature maps on the 11th and 26th channels at all timesteps are basically noise features.

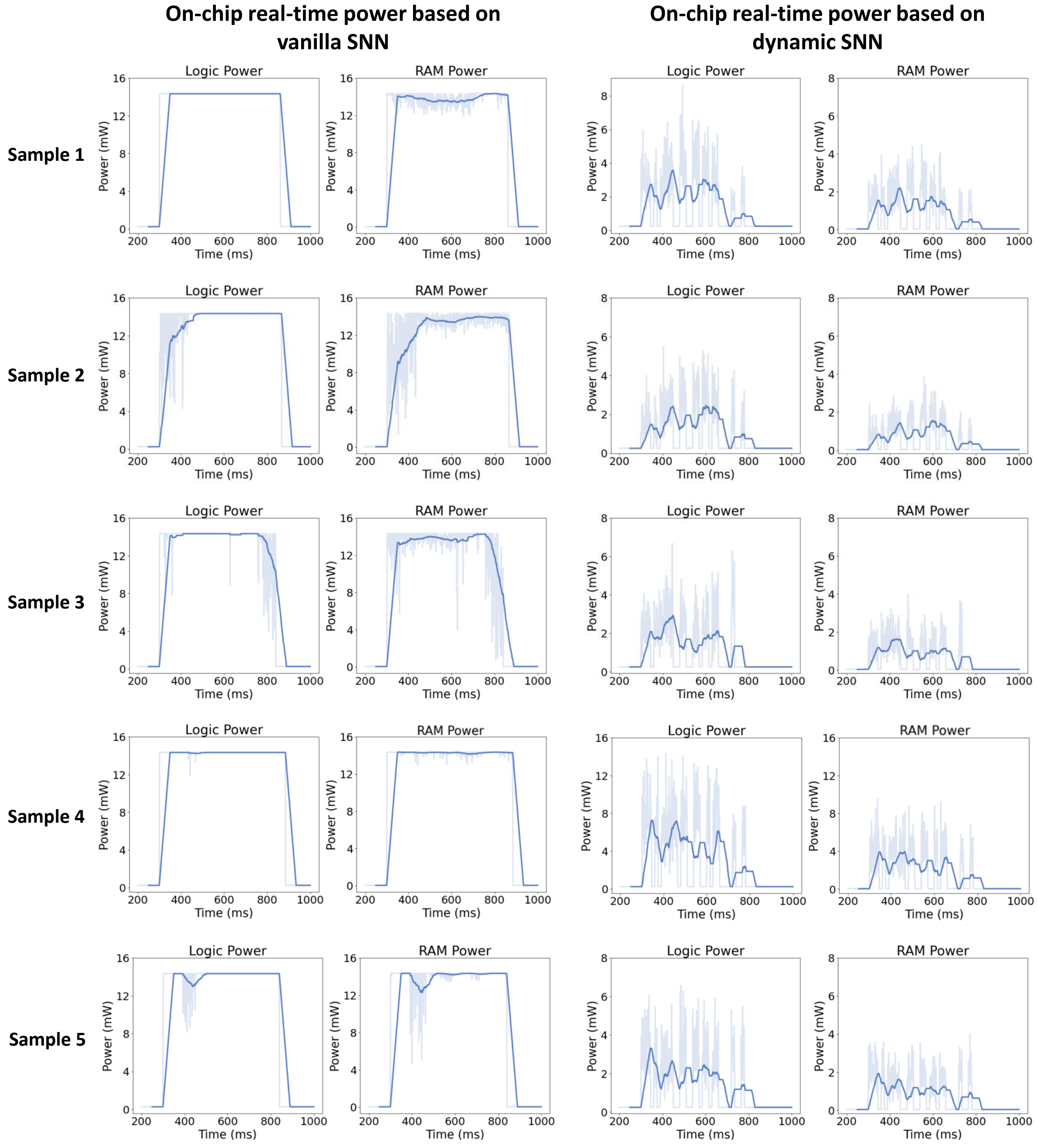

Figure S3. Impact of dynamic imbalance on power consumption. When deploying the vanilla SNN on Speck, the dynamic imbalance makes the power curve almost a straight line since the Network Spiking Firing Rate (NSFR) varies little at different moments. In contrast, deploying dynamic SNN on Speck, the power varies greatly at different moments, and the total energy consumption is significantly reduced. Specifically, Speck provides a real-time power (RAM and Logic power) monitoring module that generates data every ms. Each input sample has a duration of 540ms. To make observation easier, we average the blurred lines on the background every 20ms to obtain a clear solid line.

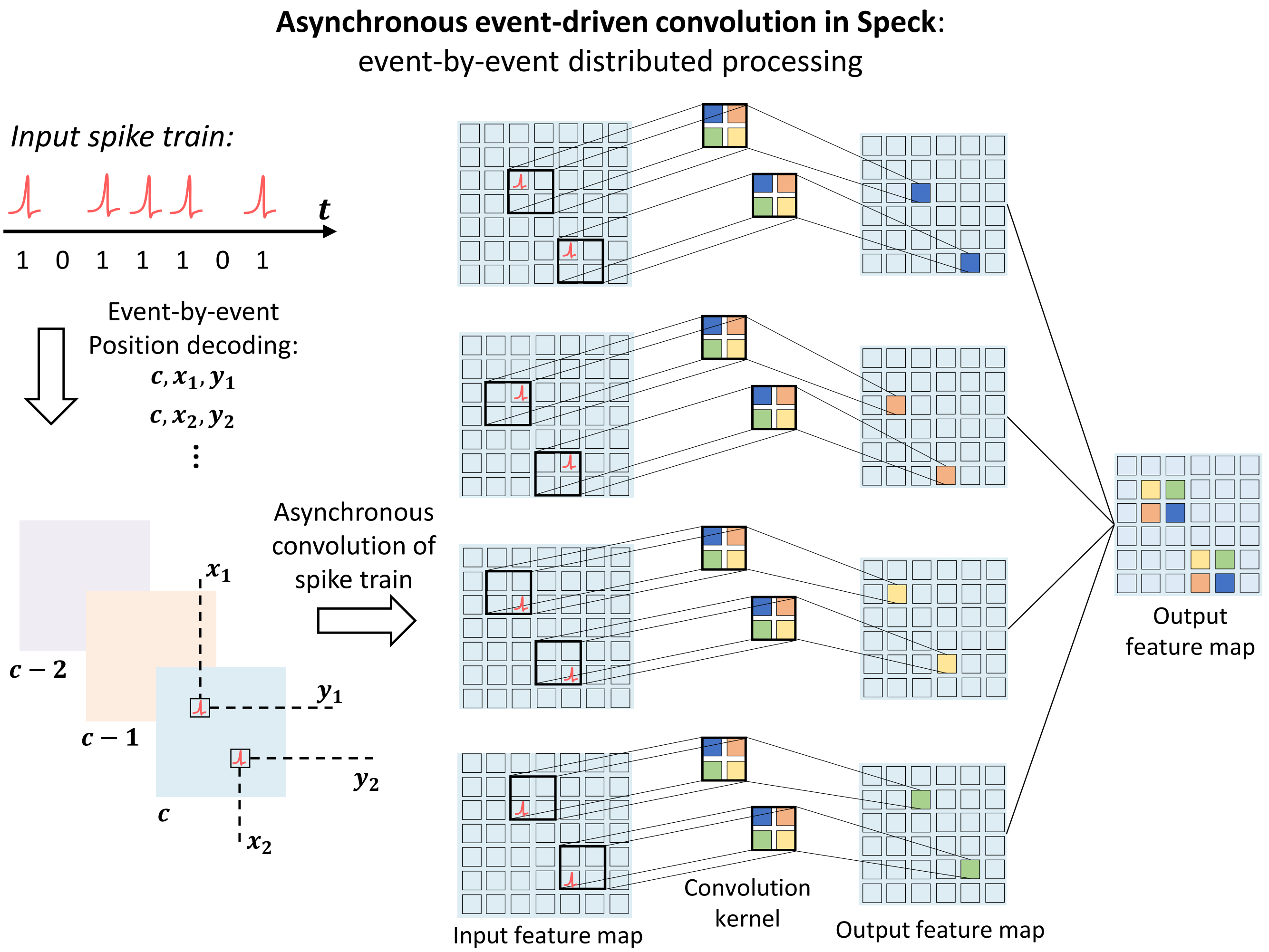

Figure S4. Asynchronous event-driven convolution in Speck. Speck processes the input event stream event-by-event, where the state of the entire system changes upon the input of a single event, thus output latency is low. Whenever an event (spike with address information) arrives at an SNN core with its positional address information, the corresponding kernel value and destination neuron position are obtained by address searching, the destination neuron states are then asynchronously updated according to the synaptic operation. Furthermore, asynchronous convolution is independent to the arrival of other input events and cores, the operation can be efficiently parallel distributed for multiple events at different positions.

Table S1. Comparison of the Speck chip with existing neuromorphic chips. Speck is a sensing-computing neuromorphic SoC, which is defined as an efficient medium-scale edge computing hardware that can meet the needs of a variety of edge visual scenarios in terms of high accuracy, low power, and low latency. Unlike other neuromorphic chips, Speck integrates a DVS with μs level temporal resolution to perceive visual information sparsely. Benefiting from fully asynchronous logic design, Speck has low rest power consumption, thus realizing the always-on profile in edge computing scenes. In contrast, classic neuromorphic chips, such as TrueNorth and Loihi, generally use a partially asynchronous design, i.e. globally asynchronous locally synchronous, or globally synchronous locally asynchronous. Compared to the earlier asynchronous neuromorphic chip Neurogrid, which used mixed-analog-digital circuits, Speck exploits a fully asynchronous digital design method that is suitable for the design of large-scale SNNs. In addition, the unique design of asynchronous convolution in Speck makes its running power consumption very low. It consumes only 0.42-15mW in typical vision application scenarios, normally only few mWs for most classical scenario applications (Normalized to a 65-nm CMOS node, and optionally to a 1.2-V supply voltage.)

**Platform**	BrainScales	SpiNNaker	Neurogrid	TrueNorth	Darwin	Loihi	Loihi-2	Tianjic	Speck
**Model**	SNN	SNN	SNN	SNN	SNN	SNN	SNN	ANN/ SNN	SNN
**Power**	1300mW	1000mW @180MHz	150mW	63-300mW	58.8mW @1.8V+70MHz	74mW	N. A.	950mW@1.2V 400mW@0.9V	0.42-15mW @1.2V
**Clock**	Partially Async	Partially Async	Async	Partially Async	Sync	Partially Async	Partially Async	Sync	Async
**Implementation Technique**	Mixed-Analog-Digital	Digital	Mixed-Analog-Digital	Digital	Digital	Digital	Digital	Digital	Digital
**Vision Sensor**	No	No	No	No	No	No	No	No	Yes
**Technology**	180nm	130nm	180nm	28nm	180nm	14nm	7nm	28nm	65nm
**Area (mm^2)**	50	102	170	430	25	60	31	14.44	30
**Number of Neuron**	512	18k	65k	1M	2048	131k	1M	40k	328k
**Number of Synapses**	128k	18M	100M	256M	4M	128M	120M (> for CNN)	9.75M	279k (up to 6.16G for CNN)
**Normalized Neuron Density (Neu/mm^2)**	79	706	2.93k	431	628	1011	374	514	11k
**Normalized Synaptic Density (Syn/mm^2)**	20k	705k	4.51M	110k	1.23M	99k	45k	125k	9.3k
**Normalized Power (process only) (mW/mm^2)**	3600	2000	415	27	163	16	-	172	0.42
**Normalized Power (process & V_{DD}) (mW/mm^2)**	-	2000	2596	11	366	6.2	-	97	0.42

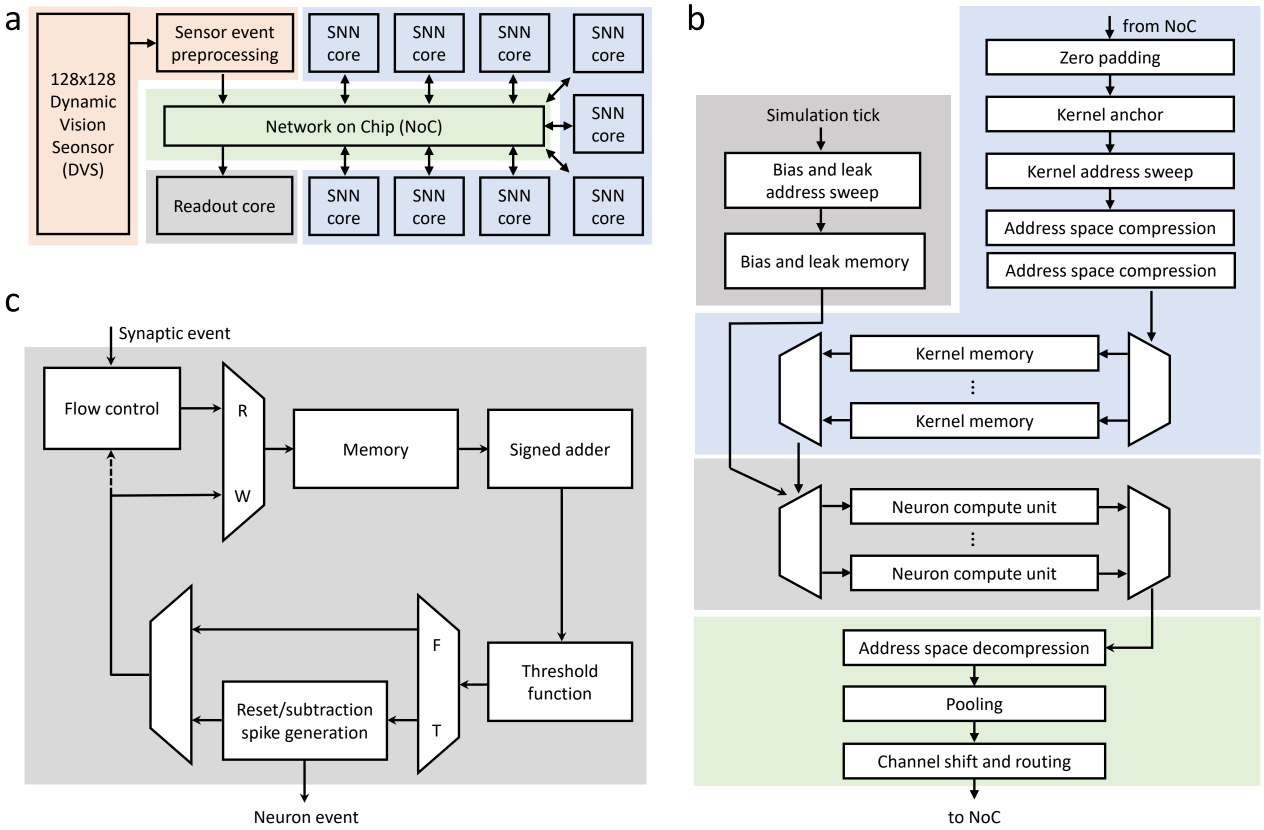

Figure S5. The Speck [4] architecture (a). The left area indicates both the 128×128 event-based vision sensor with its 2D asynchronous readout and the sensor event pre-processing pipeline. The middle area indicates the NoC responsible for all the event routing between all the components. The area indicated on the right incorporates all the nine SNN cores that handle one convolution and one pooling layer each. The SNN cores can optionally be operated as fully connected SNN layers with some restrictions. The small bottom area indicates the decision readout logic. This core enables interfacing to simple synchronous periphery. The convolution core [5] architecture (b). An event $\{c,x,y\}$ enters the convolution core pipeline, with c as the incoming channel/feature, x as the horizontal coordinate and y as the vertical coordinate. After padding, the event is now expanded to $\{c,x_p,y_p\}$. The Kernel Anchor determines the anchor in both kernel and neuron space $\{c,x_0,y_0,x_0^k,y_0^k\}$. With x_0,y_0 being the anchor in the neuron space and x_0^k,y_0^k for the kernel space. The kernel address sweep now calculates the kernel expansion in x, y and f the output channel/features to $Z * \{(c,f,x^k,y^k),(f,x,y)\}$, with Z being the synaptic fan-out. The parallel address compression packs the storage addresses compact to avoid unused storage gaps for the neuron $(f,x,y) => n_{\text{comp}}$ and kernel $(c,f,x^k,y^k) => k_{\text{comp}}$. Depending on the core, the kernel memory is split into one or multiple memory blocks for parallel access. The kernel value is read from the storage address k_{comp}, $\{w,n_{\text{comp}}\}$ with w being the signed 8-bit synaptic weight. On a simulation tick, the bias/leak sweep will generate a pair of $\{b_{\text{comp}},n_{\text{comp}}\}$ for every active neuron, the address b_{comp} gets read in the bias/leak memory and forwarded as $\{w,n_{\text{comp}}\}$ with the kernel events to the neuron. Depending on the core, the neuron unit is split into one or multiple parallel compute units. The address space decompression turns the $\{n_{\text{comp}}\}$ back to $\{f,x,y\}$. The sum pooling operates on the same event structure $\{f,x_s,y_s\}$. And the Channel shift and routing prepare it for routing $S * \{d_x,f_s,x_s,y_s\}$ with S being the source fan-out of 1 or 2, d_x corresponding to the destination id and f_s being the arithmetically shifted destination channel. The neuron compute unit [5] (c). It uses in-memory-controller compute to model the LIF neuron model. The flow control at the input ensures that the controller always has a bubble and is therefore deadlock-free. The signed 16-bit neuron state variable gets read, modified by the signed synaptic or bias input, compared and written back. In case a threshold condition is met, the $\{n_{\text{comp}}\}$ is sent out to indicate the corresponding neuron spiked. The above-threshold condition can both trigger a subtraction operation or a reset to a fixed value of the corresponding neuron state variable. The state variable cannot cross a configured lower bound and will be clamped to that value in case any operation brings the variable below it.

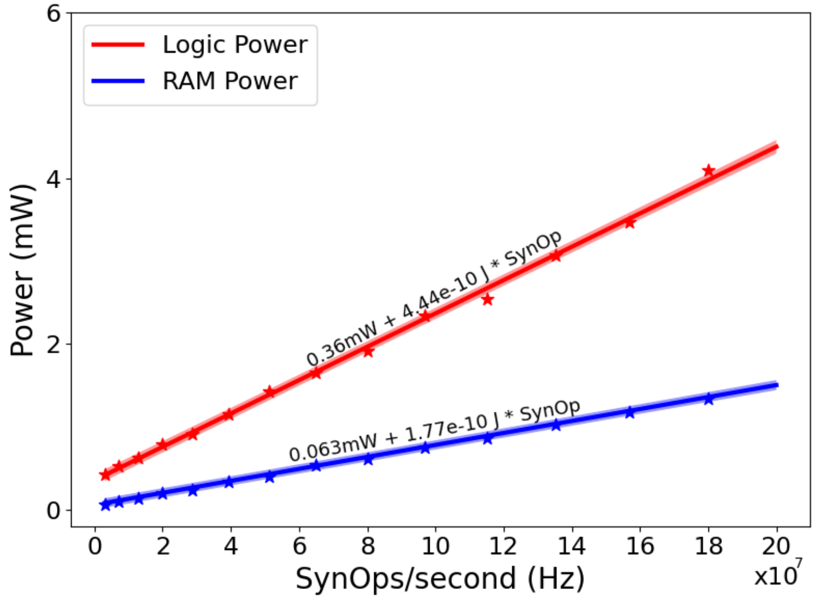

Figure S6. Unit power measurement based on the linear relationship between SynOps/s and power. Synaptic Operations (SynOp) is the basic unit of energy consumption assessment in Speck, which is defined as all the steps involved in the life-cycle of a spike arriving at a layer until it updates the neuron states and generates a spike if applied. A SynOp includes the following steps: logic → Kernel RAM → logic → Neuron RAM Read → Neuron RAM Write → Logic. Thus, whenever a spike arrives at a core, the power consumption can be roughly divided into two parts: RAM power and Logic power. We send random events with fixed firing rates to a specific layer to trigger computations, and measure the RAM and Logic power of the chip. Then, we get a curve of power-SynOps, and the results are shown in the figure. The intercept and slope of the fitted straight line represent resting power and energy consumption of a single SynOp, respectively.

	**Resting Power (mW)**	**Running Power (pJ/SynOp)**
**Logic**	0.36	444 ± 2.7
**RAM**	0.06	177 ± 1.6

Table S2. Power consumption of Speck with 9 SNN cores. The processor resting power is about 0.42mW. The data in this table are obtained from Fig. S6.

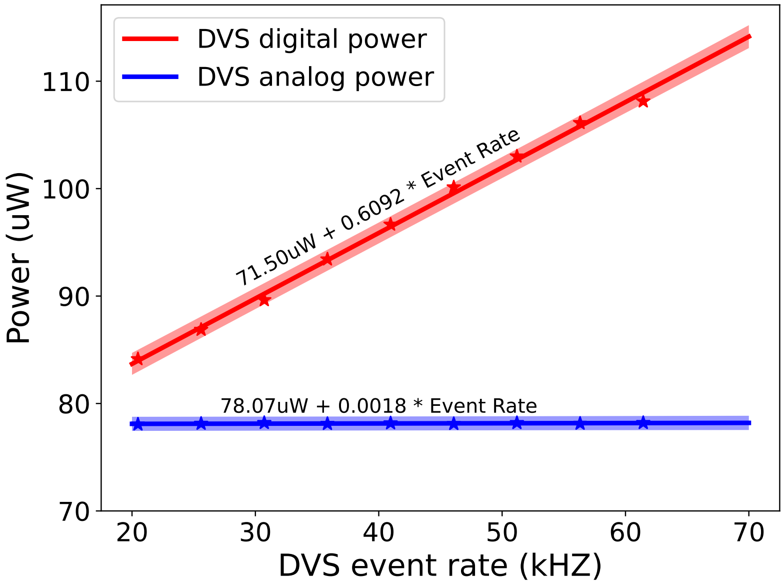

Figure S7. DVS power consumption measurement based on the linear relationship between event rate and power.

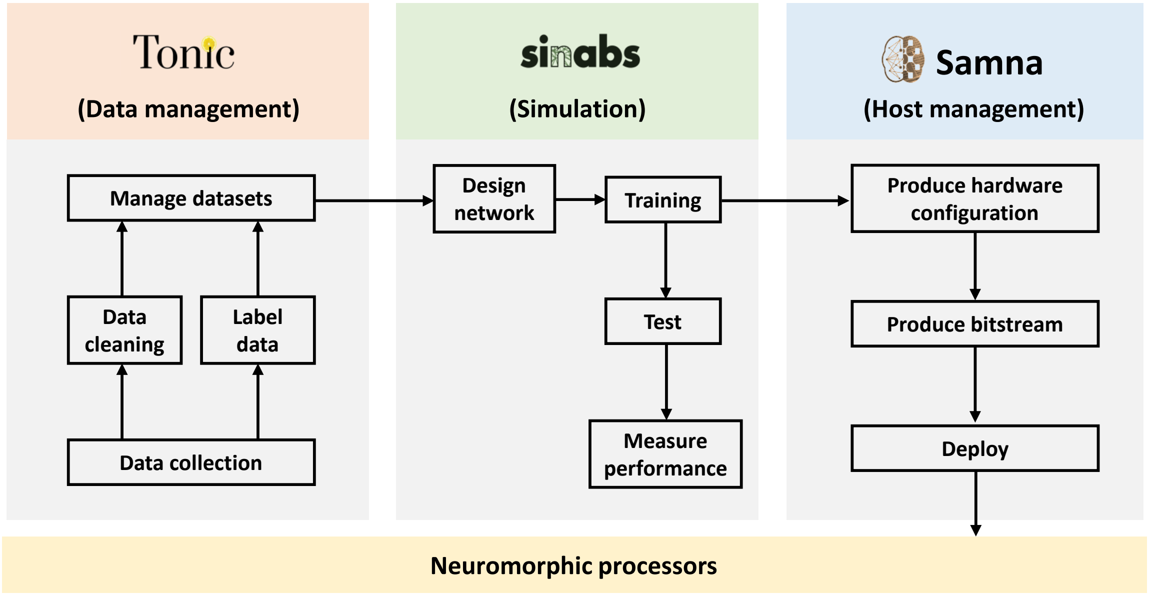

Figure S8. Speck's complete software toolchain for efficient neuromorphic application development. Data management: The Tonic software can help the user efficiently works on the asynchronous event data. Tonic caters to both the event-based world that works directly with events or time surfaces as well as to more conventional frameworks which might convert events into dense representations in one way or another. Simulation: Sinabs is a PyTorch-based SNN development framework, which enables the design, training, and evaluation of SNNs as well as simulating the hardware-compatible models. Host management: Samna is developed towards efficiency and user friendly. A set of Python APIs is available with the core running in C++, and it is possible to work with neuromorphic devices in a professional and elegant manner. Samna also features an event stream filter system that allows real-time, multi-branch processing of the event stream coming in or out from the device. With an integration of a just-in-time compiler in Samna, the flexibility of this filter system has been taken to an even higher dimension, which supports adding users' defined filter functions at run-time to meet the requirements of different scenarios.

sinabs	docs: https://sinabs.ai<br>github: https://github.com/synsense/sinab
samna	docs: https://synsense-sys-int.gitlab.io/samna/
Tonic	docs: https://tonic.readthedocs.io/en/latest/?badge=latet<br>github: https://github.com/synsense/sinab

Table S3. Open source URLs related to the Speck software toolchain.

	TensorFlow	PyTorch	Norse	SNNTorch	Speck Tools
Asynchronous simulation	✗	✗	✗	✗	✓
CPU/GPU/TPU acceleration	✓	✓	✓	✓	✓
Spiking neuron modelling	✗	✗	✓	✓	✓
Open source	✓	✓	✓	✓	✓
SNN direct training	✗	✗	✓	✓	✓
ANN/SNN parameter quantization	✗	✗	✗	✗	✓
Support mixed-signal SNN development	✗	✗	✗	✗	✓
Neuromorphic hardware compatible simulation	✗	✗	✗	✗	✓
Support convolutional dedicated neuromorphic chips	✗	✗	✗	✗	✓

Figure S9. The comparison between Speck tools and existing general AI development tools. The Speck tools comprise of softwares that can completely support the hardware-dedicated SNN design and testing. This includes data management tools, network design and training tools, simulation tools, and deployment tools. Compared to existing AI tools, Speck aims to provide an efficient way of designing and simulating the network working in a fully streaming, event-driven fashion.

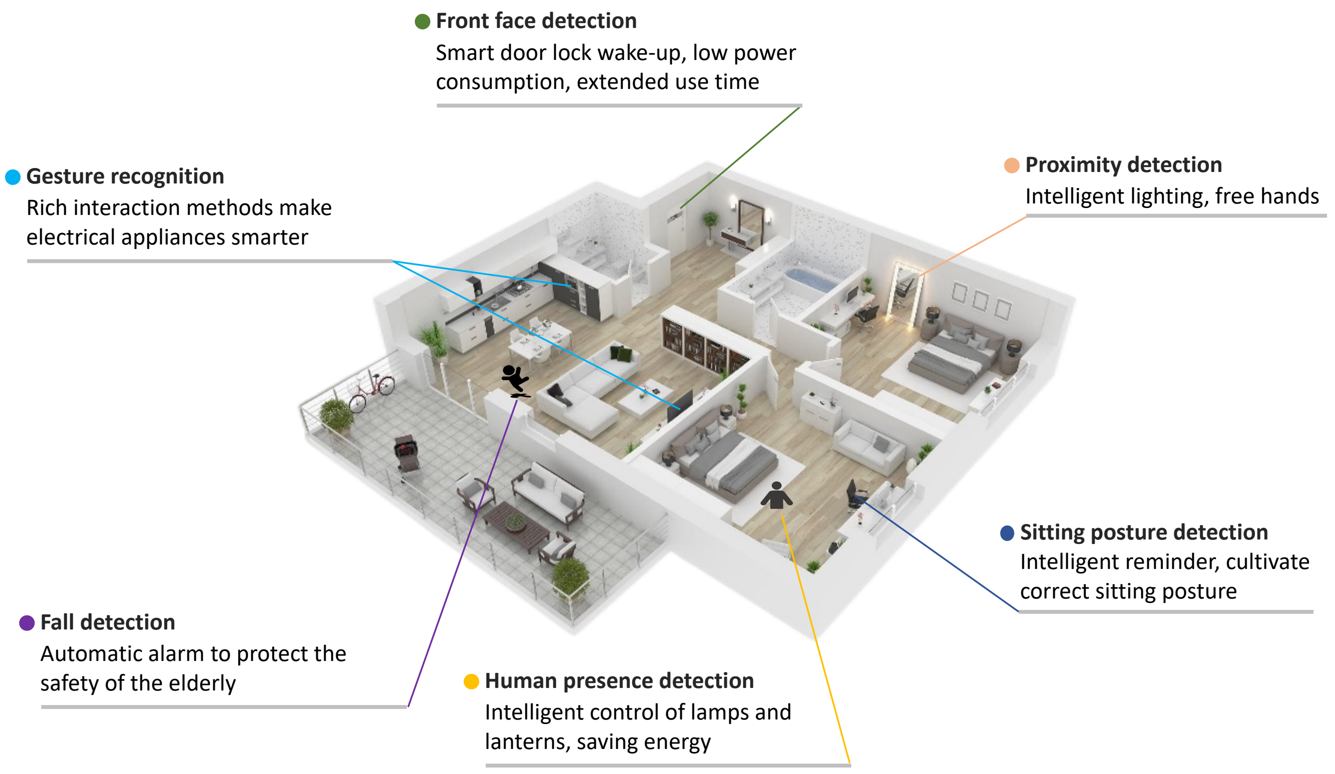

Figure S10. Complete smart home solution based on Speck. Some typical application examples of the Speck in the smart home are shown in the figure, e.g., front face detection, gesture recognition, and proximity detection can be used to trigger certain electronic furniture like lamps and curtains. Moreover, some safety monitoring applications, such as presence detection and fall detection, can be used to send notices to households. We have provided some movies of the Speck being used in typical application scenarios as supplementary material.

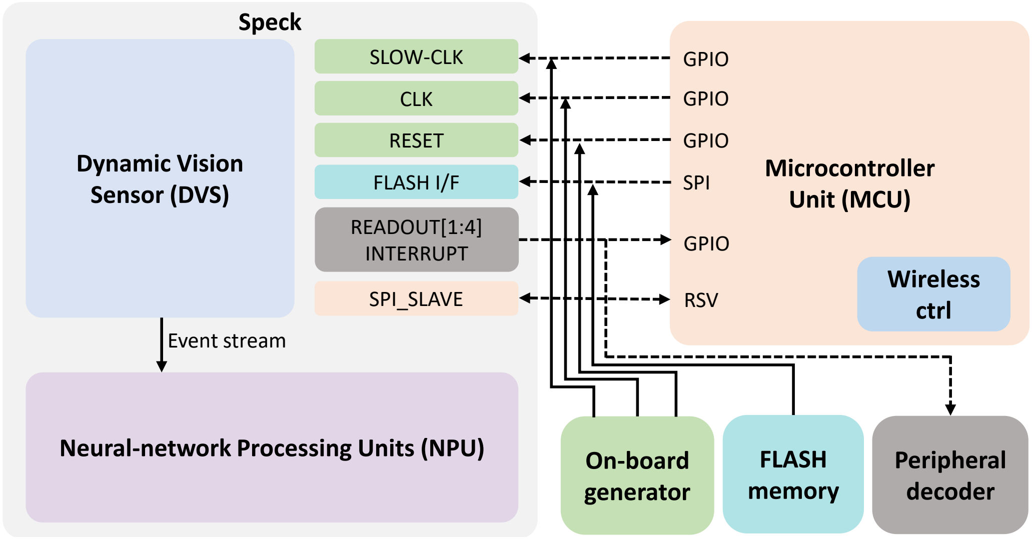

Figure S11. Demonstration example of a Speck System communicating with an external Micro-controller Unit (MCU) device. Speck can be configured to perform bidirectional communication with external devices via multiple interfaces. SLOW-CLK: external slow clock signal used to drive readout. CLK: External clock signal for data transmission. RESET: reset signal. FLASH: external memory used to preserve the chip configuration. READOUT: readout output interrupt signal. SPI SLAVE: used for data transmission.

Hyper-parameter	Gesture/Gait-day/Gait-night
Temporal resolution dt	15
Simulation timestep T	36
Spatial resolution	32×32
Spike firing threshold	0.3
Temporal mask ratio of dynamic SNN	0.5
Batchsize	64
Epochs	100
Weight decay	1e-4
Learning rate	5e-4
Learning algorithm	STBP
Loss function	CrossEntropyLoss
Optimizer	Adam

Table S4. Training Hyper-parameter setting.

Table S5. Comparison with prior works. The numbers in brackets denote the performance improvement over the re-implementation baselines. Throughout all ablation experiments, the only variable was whether the proposed module was plugged into the vanilla SNNs. Considering the requirement of network scale during hardware deployment, on Gesture, Gait-day, and Gait-night, we limit the size of the baseline model to focus on evaluating the gain of the proposed dynamic framework on lightweight models.

Dataset	Methods	Input time window (ms)	Network Average Spiking Firing Rate (NASFR)	Acc. (%)
DVS128 Gesture [6]	Recurrent CNN [6]	120	-	92.6
	Slayer [10]	1500	-	93.6
	STBP-tdBN [11]	1200	0.15	96.9
	SEW-ResNet [12]	6000	0.05	97.9
	Spiking DS-ResNet [13]	-	-	97.3
	LIF-SNN [14]	540	0.18	89.9
	+ AR (Ours)		0.05	95.1 (+5.2)
	LIF-SNN [15]	6000	0.07	94.8
	+ AR (Ours)		0.04	97.7 (+2.9)
DVS128 Gait-day [7]	EV-Gait [7]	4400	-	89.9
	EV-3DGraph [8]	4400	-	94.9
	EV-3DGraph [8]	200	-	38.5
	LIF-SNN [14]	540	0.23	86.7
	+ AR (Ours)		0.06	90.8 (+4.1)
	LIF-SNN [15]	4400	0.05	89.9
	+ AR (Ours)		0.03	92.6 (+2.7)
DVS128 Gait-night [8]	EV-3DGraph [8]	5500	-	96.0
	LIF-SNN [14]	540	0.18	95.7
	+ AR (Ours)		0.14	98.8 (+3.1)
HAR-DVS [9]	Event-based Transformer [9]	$T = 8$	-	51.2
	ResNet-18 [9]		-	49.2
	X3D [9]		-	45.8
	Res-SNN-18 [12]	$T = 8$	0.19	45.8
	+ AR (Ours)		0.17	46.7 (+ 0.9)

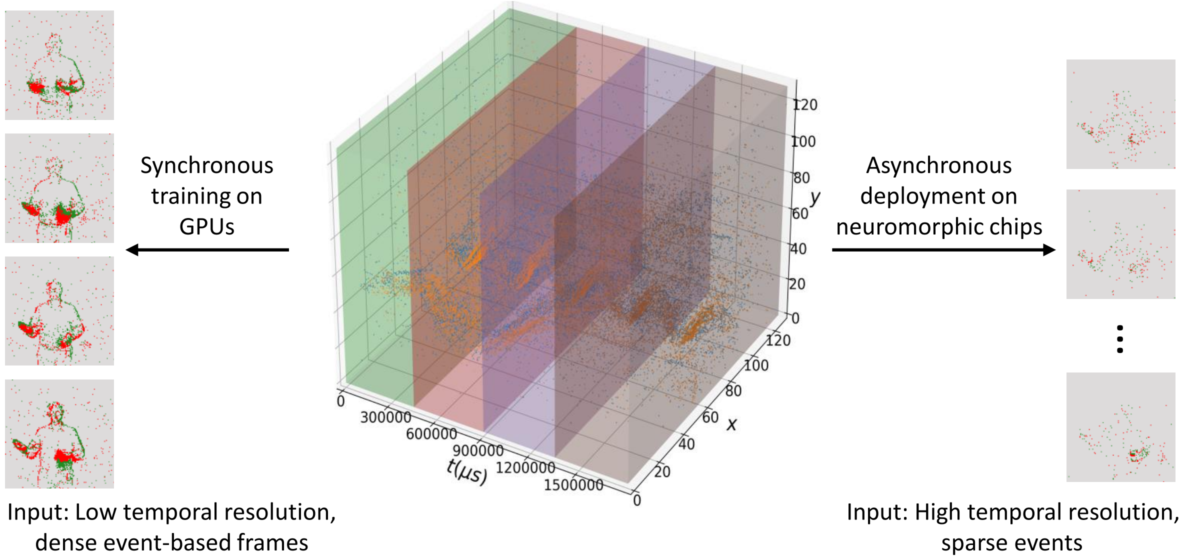

Figure S12. **Synchronous training and asynchronous deployment.** To assure the accuracy of event-based vision tasks, when using the SNN algorithm, the event stream is generally aggregated into an event-based frame sequence, and then sent to the network for training. As shown in the left part of the figure, after the event stream is converted into a frame sequence, the amount of information contained in each frame input increases, which helps to improve task accuracy. However, after the model trained on the GPU is deployed on the neuromorphic chip, the input received is one event after another. As shown in the right part of the figure, the input information actually received by the neuromorphic chip is sparse. This kind of synchronous training on the GPU and asynchronous deployment on the neuromorphic chip will inevitably reduce the task accuracy due to the difference in input data.

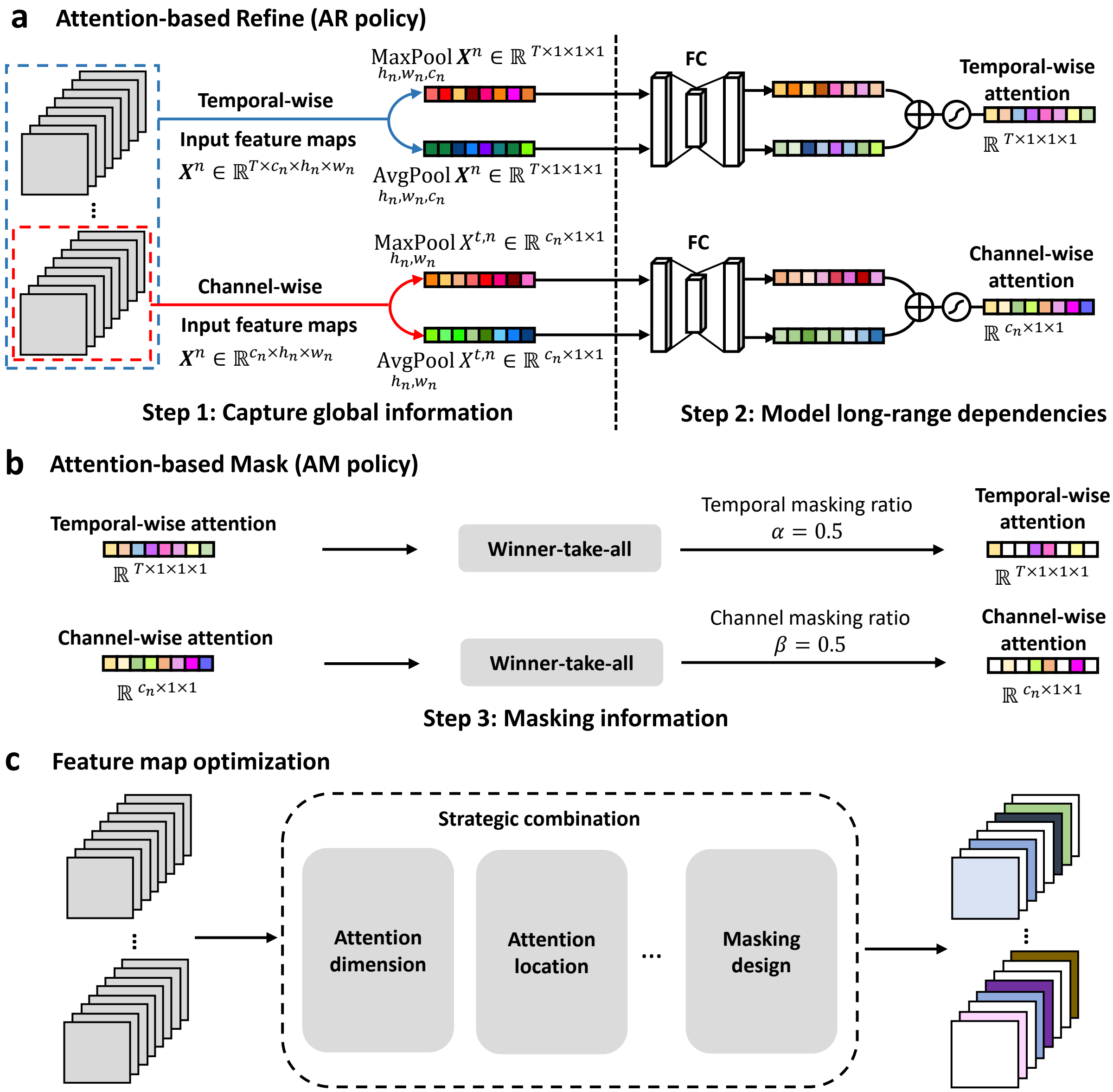

Figure S13. **Attention-based dynamic module design.** **a**, Step1, capture global information. Step2, model long-range dependencies. **b**, masking information. **c**, Feature map optimization. Note, the proposed dynamic framework contains a huge design space in each step [16,17]. In practice, we can make special designs according to the needs of application scenarios in terms of accuracy, parameter, and computation workload, etc.

Supplementary References

[1] Huang, Ziyuan, Shiwei Zhang, Liang Pan, Zhiwu Qing, Mingqian Tang, Ziwei Liu, and Marcelo H. Ang Jr. “TAda! Temporally-Adaptive Convolutions for Video Understanding." In *International Conference on Learning Representations*, 2022.

[2] Yao, Man, Jiakui Hu, Guangshe Zhao, Yaoyuan Wang, Ziyang Zhang, Bo Xu, and Guoqi Li. “Inherent redundancy in spiking neural networks." In *Proceedings of the IEEE/CVF International Conference on Computer Vision*, pp. 16924-16934. 2023.

[3] Wang, Xiaolong, Ross Girshick, Abhinav Gupta, and Kaiming He. “Non-local neural networks." In *Proceedings of the IEEE Conference on Computer Vision and Pattern Recognition*, pp. 7794-7803. 2018.

[4] Tugba Demirci, Ning Qiao, Sheik Sadique, and Ole Richter. “Event-driven integrated circuit having interface system." *WIPO Patent App* No. PCT/CN2021/088143.IPN.WO2022/221994A1. 2022.

[5] Richer Ole, Qian Liu, Ning Qiao, and Sadique Sadique. “Event-driven spiking convolutional neural network." *WIPO Patent App* No. PCT/EP2020/059798.IPN.WO2020/207982A1. 2020.

[6] Amir, Arnon, Brian Taba, David Berg, Timothy Melano, Jeffrey McKinstry, Carmelo Di Nolfo, Tapan Nayak et al. “A low power, fully event-based gesture recognition system." In *Proceedings of the IEEE Conference on Computer Vision and Pattern Recognition*, pp. 7243-7252. 2017.

[7] Wang, Yanxiang, Bowen Du, Yiran Shen, Kai Wu, Guangrong Zhao, Jianguo Sun, and Hongkai Wen. “EV-gait: Event-based robust gait recognition using dynamic vision sensors." In *Proceedings of the IEEE/CVF Conference on Computer Vision and Pattern Recognition*, pp. 6358-6367. 2019.

[8] Wang, Yanxiang, Xian Zhang, Yiran Shen, Bowen Du, Guangrong Zhao, Lizhen Cui, and Hongkai Wen. “Event-stream representation for human gaits identification using deep neural networks." *IEEE Transactions on Pattern Analysis and Machine Intelligence* 44, no. 7 (2021): 3436-3449.

[9] Wang, Xiao, Zongzhen Wu, Bo Jiang, Zhimin Bao, Lin Zhu, Guoqi Li, Yaowei Wang, and Yonghong Tian. “Hardvs: Revisiting human activity recognition with dynamic vision sensors." *arXiv preprint* arXiv:2211.09648 (2022).

[10] Shrestha, Sumit B., and Garrick Orchard. “Slayer: Spike layer error reassignment in time." *Advances in Neural Information Processing Systems* 31 (2018).

[11] Zheng, Hanle, Yujie Wu, Lei Deng, Yifan Hu, and Guoqi Li. “Going deeper with directly-trained larger spiking neural networks." In *Proceedings of the AAAI conference on artificial intelligence*, vol. 35, no. 12, pp. 11062-11070. 2021.

[12] Fang, Wei, Zhaofei Yu, Yanqi Chen, Tiejun Huang, Timothée Masquelier, and Yonghong Tian. “Deep residual learning in spiking neural networks." *Advances in Neural Information Processing Systems* 34 (2021): 21056-21069.

[13] Feng, Lang, Qianhui Liu, Huajin Tang, De Ma, and Gang Pan. “Multi-level firing with spiking ds-resnet: Enabling better and deeper directly-trained spiking neural networks." *arXiv preprint* arXiv:2210.06386 (2022).

[14] Yao, Man, Huanhuan Gao, Guangshe Zhao, Dingheng Wang, Yihan Lin, Zhaoxu Yang, and Guoqi Li. “Temporal-wise attention spiking neural networks for event streams classification." In *Proceedings of the IEEE/CVF International Conference on Computer Vision*, pp. 10221-10230. 2021.

[15] Fang, Wei, Zhaofei Yu, Yanqi Chen, Timothée Masquelier, Tiejun Huang, and Yonghong Tian. “Incorporating learnable membrane time constant to enhance learning of spiking neural networks." In *Proceedings of the IEEE/CVF International Conference on Computer Vision*, pp. 2661-2671. 2021.

[16] Han, Yizeng, Gao Huang, Shiji Song, Le Yang, Honghui Wang, and Yulin Wang. “Dynamic neural networks: A survey." *IEEE Transactions on Pattern Analysis and Machine Intelligence* 44, no. 11 (2021): 7436-7456.

[17] Guo, Meng-Hao, Tian-Xing Xu, Jiang-Jiang Liu, Zheng-Ning Liu, Peng-Tao Jiang, Tai-Jiang Mu, Song-Hai Zhang, Ralph R. Martin, Ming-Ming Cheng, and Shi-Min Hu. "Attention mechanisms in computer vision: A survey." *Computational Visual Media* 8, no. 3 (2022): 331-368.
